# Clinical and radiological outcome following treatment of displaced lateral clavicle fractures using a locking compression plate with lateral extension: a prospective study

**DOI:** 10.1186/1471-2474-15-380

**Published:** 2014-11-19

**Authors:** Marc Beirer, Sebastian Siebenlist, Moritz Crönlein, Lukas Postl, Stefan Huber-Wagner, Peter Biberthaler, Chlodwig Kirchhoff

**Affiliations:** Department of Trauma Surgery, Klinikum rechts der Isar, Technical University of Munich, Ismaningerstrasse 22, Munich, 81675 Germany

**Keywords:** Lateral clavicle fracture, Distal clavicle fracture, Locking compression plate, Superior anterior clavicle plate with lateral extension, Instability

## Abstract

**Background:**

Treatment of lateral fractures of the clavicle is challenging and has been controversially discussed for a long time due to high non-union rates in non-operative treatment and high complication rates in surgical treatment. Acromioclavicular joint instability due to the injury of the closely neighbored coraco-clavicular ligaments can result in a cranialization of the medial clavicle shaft. A recently developed implant showed a promising functional outcome in a small collective of patients.

**Methods:**

In this prospective study, 20 patients with a mean age of 40.7 ± 11.3 years with a dislocated fracture of the lateral clavicle (Jäger&Breitner I-III, Neer I-III) were enrolled. All patients were surgically treated using the locking compression plate (LCP) for the superior anterior clavicle (Synthes®). Functional outcome was recorded using the Munich Shoulder Questionnaire (MSQ) allowing for qualitative self-assessment of the Shoulder Pain and Disability Index (SPADI), of the Disability of the Arm, Shoulder and Hand (DASH) score and of the Constant Score. Acromioclavicular joint stability was evaluated using the Taft-Score.

**Results:**

The mean follow-up was 14.2 ± 4.0 months. The mean MSQ was 87.0 ± 7.4 points, the mean SPADI 91.1 ± 11.3 points, the mean DASH score 7.6 ± 7.3 points and the mean normative age- and sex-specific Constant Score 85.6 ± 8.0 points. The mean Taft Score resulted in 10.7 ± 1.0 points. The mean Taft Score in lateral clavicular fractures with fracture gap between the coracoclavicular ligaments in combination with a rupture of the conoid ligament (J&B II a, Neer II B; n =11) was with 10.3 ± 0.9 points significantly lower than the mean Taft Score of all other types of lateral clavicle fractures (J&B I, II b, III; n =9) which resulted in 11.3 ± 0.9 points (p < 0.05).

**Conclusions:**

The Synthes® LCP superior anterior clavicle plate allows for a safe stabilization and good functional outcome with high patient satisfaction in fractures of the lateral clavicle. However, in fractures type Jäger&Breitner II a, Neer II B a significant acromioclavicular joint instability was observed and additional reconstruction of the coracoclavicular ligaments should be performed.

**Trial registration:**

ClinicalTrials.gov NCT02256059. Registered 02 October 2014.

**Electronic supplementary material:**

The online version of this article (doi:10.1186/1471-2474-15-380) contains supplementary material, which is available to authorized users.

## Background

Fractures of the lateral end of the clavicle account for approximately 18% of all clavicular fractures [1] and occur most commonly in young adults following sports injury and less frequently following simple falls [2]. Non-operative treatment of lateral clavicle fractures (Neer type II) results in a non-union rate of 33% [3], whereas surgical treatment leads to a union rate of up to 95% [4]. Previous operative approaches such as osteosynthesis using the hook plate or k-wires led to an increased complication rate of up to 22% [3], most likely due to biomechanically insufficient stabilization of the short, metaphyseal fracture fragment. Therefore treatment of lateral clavicle fractures was controversially discussed for a long time. Recently several authors reported promising clinical and radiological results using a new, precontoured, locking compression plate with lateral extension, the so-called LCP superior anterior clavicle plate (Depuy-Synthes®, 4528 Zuchwil, Switzerland), usable for both internal fixation of lateral clavicle fractures [5, 6] and operative management of clavicular non-union [7]. However, up to now, there are no studies focusing on long term follow-up.

Another crucial issue concerning bony union and functional results of surgically treated distal clavicle fractures is the potential correlation to the integrity of the acromio-clavicular (AC) complex. Besides integrity of the coraco-clavicular (CC) and AC ligaments, the weight of the arm, scapular rotation and muscle forces (latissimus dorsi muscle, pectoralis major and minor muscle, trapezius muscle) have been reported to impair bony union [8]. Several authors reported good functional and radiological results after plate fixation of distal clavicle fractures J&B II a in combination with reconstruction of the CC ligaments [6, 9] and therefore it must be considered whether specific fracture types require more than single plate fixation.

In summary the aim of this prospective study was to evaluate the clinical and radiological outcome after a mean follow-up of one year and especially to assess the correlation between fracture type and postoperative AC instability using the Taft Score [10].

## Methods

### Patients

The study protocol was approved by the local ethics committee (Ethics Committee of the medical faculty, Klinikum rechts der Isar, Technical University of Munich, Germany; study number 5536/12). Patients suffering from a dislocated fracture of the lateral clavicle presenting at our emergency department were identified and prospectively enrolled in the study after informed consent. All patients gave their written consent to publish their data in Table 1. All fractures were classified according to the Jäger&Breitner (J&B) classification [11] as an extension of the Neer classification [8]. Preoperative standard radiographs of the clavicle (anterior-posterior perpendicular to cassette and anterior-posterior 30 degree angle cephalad) were performed. Patients with a history of any other pathology such as preexisting rotator cuff tear, gleno-humeral instability, glenohumeral osteoarthritis (> Samilson I), AC joint instability, AC osteoarthritis, calcifying tendonitis, biceps pathology or signs of cervical root symptoms were excluded from the study. Written informed consent was obtained from each patient.

**Table 1:** **Patient demographics and outcomes**

Number	Age	Sex	J&B	Plate removed	Follow up (months)	MSQ	SPADI	DASH score	Rel. CS	Taft score	Injury mechanism	Dominant side injured	Delay trauma-repair (days)	Time length for FFR (days)
1	39	M	IIa	Yes	13	91	97	7	93	11	vehicle accident	Yes	10	35
2	26	M	I	Yes	16	91	98	4	90	12	bike accident	Yes	3	26
3	33	W	I	Yes	12	72	68	26	84	10	bike accident	No	5	44
4	35	M	IIa	Yes	16	86	92	9	87	11	ski accident	Yes	4	31
5	59	M	IIa	Yes	14	98	99	0	100	11	bike accident	No	2	37
6	29	M	IIa	No	12	86	86	7	81	10	jogging accident	No	2	23
7	45	M	IIa	No	12	89	97	2	84	10	bike accident	No	7	31
8	28	M	III	No	12	93	98	2	87	12	jogging accident	Yes	6	37
9	47	W	III	No	13	96	100	0	100	12	simple fall	No	6	46
10	31	W	I	Yes	12	86	95	8	84	11	bike accident	Yes	3	42
11	37	M	IIa	No	13	84	89	13	85	11	ski accident	Yes	2	48
12	46	M	I	Yes	15	90	95	1	85	12	ski accident	No	6	39
13	53	M	IIa	No	12	87	95	6	85	9	bike accident	Yes	1	38
14	36	M	IIa	Yes	19	94	98	0	90	11	bike accident	Yes	5	34
15	39	M	IIa	Yes	21	92	100	1	83	11	bike accident	No	4	41
16	21	W	IIb	No	14	83	88	14	87	12	vehicle accident	Yes	4	28
17	52	W	IIa	No	12	70	58	20	65	9	simple fall	Yes	8	44
18	54	M	IIb	No	7	77	77	18	75	10	bike accident	Yes	9	39
19	61	W	IIa	No	26	82	92	8	76	9	simple fall	No	1	43
20	43	M	I	No	12	92	99	6	91	11	bike accident	Yes	2	26

Jäger&Breitner (J&B); Munich Shoulder Questionnaire (MSQ); Shoulder Pain and Disability Index (SPADI); Disability of the Arm, Shoulder and Hand (DASH) score; relative Constant Score (Rel. CS); full functional recovery (FFR).

### The implant

The LCP superior anterior clavicle plate with lateral extension (Depuy-Synthes®, 4528 Zuchwil, Switzerland) is an anatomically precontoured fixation system with three to eight medial shaft holes for 3.5 mm locking or 3.5 mm cortex screws and six lateral 2.7 mm divergent locking or 2.4 mm cortex screws.

### Surgical technique and rehabilitation

All patients underwent surgical intervention with open reduction and internal fixation (ORIF) in beachchair position with the affected arm in a mobile position. A transverse skin incision was made upon the clavicle with lateral extension to the lateral edge of the acromion. The AC joint capsule was not incised. After sharp dissection of the periosteum and debridement of fracture hematoma, the fracture was sparingly exposed. To gain anatomical reduction the fracture was temporarily reduced using two k-wires as temporary arthrodesis of the AC joint or using a reduction forceps. The position was checked using fluoroscopy. The AC joint was located by temporary insertion of a needle. The plate was centered onto the clavicular shaft and after confirmation of correct plate positioning in fluoroscopy, screw holes were consecutively drilled. If necessary inferior butterfly fragments of the CC-insertion were fixed using a cerclage (FiberWire 2, Arthrex, Naples, USA).

Post surgery the arm was immobilized in a sling (Medi Sling, Medi SAK, Bayreuth, Germany) and patients started physiotherapy on the first postoperative day following a standard rehabilitation protocol: abduction and flexion were restricted to 90° for the first six weeks. With decreasing pain, this training was progressed with strengthening exercises of the rotator cuff and shoulder muscles. Return to sportive activity of the upper extremities was allowed after another 6 weeks.

### Follow-up

Clinical and radiological outcome was assessed during routine follow-up examinations 6, 12, 36 weeks and one year after surgery in our outpatient clinic. The Munich Shoulder Questionnaire (MSQ) presents an universally applicable instrument for the self-assessment of the shoulder function. It was developed for an effective follow-up of shoulder patients allowing for a quantitative assessment of the Shoulder Pain and Disability Index (SPADI), the Disability of the Arm, Shoulder and Hand (DASH) score and the Constant Score. The MSQ has been validated previously and its accuracy and effectiveness for follow-up assessment was demonstrated [12, 13]. Original Constant Score values were used to calculate a normative age- and sex-specific Constant Score (relative Constant Score) according to Gerber et al. [14]. AC joint stability was assessed by determining the Taft score which includes subjective, objective and radiologic criteria [10].

### Statistics

Data is given in terms of the arithmetic mean ± standard deviation and the range in brackets. The results were compared by calculating the Wilcoxon rank-sum test. A p-value <0.05 determined significance. Statistics were calculated using commercially available programs (SigmaStat 3.1, SigmaPlot 8.02, Systat Software Inc., Chicago, USA). A p-value less than 0.05 was considered as statistically significant.

## Results

### Demographics and fracture morphology

Between June 2011 and September 2013, 20 dislocated fractures of the lateral clavicle in 20 patients (14 men, 6 women) with a mean age of 40.7 ± 11.3 years (21–61 years) were enrolled in the study and surgically treated using the Synthes® LCP superior anterior clavicle plate in a prospective clinical trial (see Table 1). The mean interval between surgery and follow-up was 14.2 ± 4.0 months (7–26 months). According to the Jäger&Breitner (J&B) classification [11] 5 patients had a type I, 11 a type II a, 2 a type II b and 2 a type III fracture.

### Surgery characteristics

The skin incisions had an average length of 5.1 ± 0.8 cm (4–7 cm). A 3-hole plate was implanted in 6, a 4-hole plate in 8, a 5-hole plate in 5 cases and a 6-hole plate in 1 case. The shaft holes were placed with at least one 3.5 mm locking screw. The plate size and the number of implanted screws depended on the fracture pattern especially on the medial extent of the fracture. On average 5 ± 1 holes (4–6 holes) of the lateral extension were placed with 2.7 mm locking screws. All four surgeons (CK, PB, SH, SS) who performed the procedure were experienced upper extremity surgeons. CK performed surgery in 8, PB in 5, SH in 3 and SS in 4 cases. Surgery had an average duration of 89.2 ± 26.7 min (46–127 min), mean dose area product as degree of intraoperative fluoroscopy was 30 ± 26 cGycm^2^ (6,69-113,31 cGycm^2^).

### Complications

There were no major complications such as wound-healing problems, infections, implant failures or revision surgeries to be reported. In 9 of 20 patients the implant was removed on average 17.6 ± 2.7 months (range 13–22 months) after surgery. Main reasons for hardware removal were either the patients’ explicit request (n = 4), implant-associated pain while carrying a heavy backpack (n = 3) or sensitivity to weather changes (n = 2).

### Patient reported and radiological outcomes

**Figure 1:**
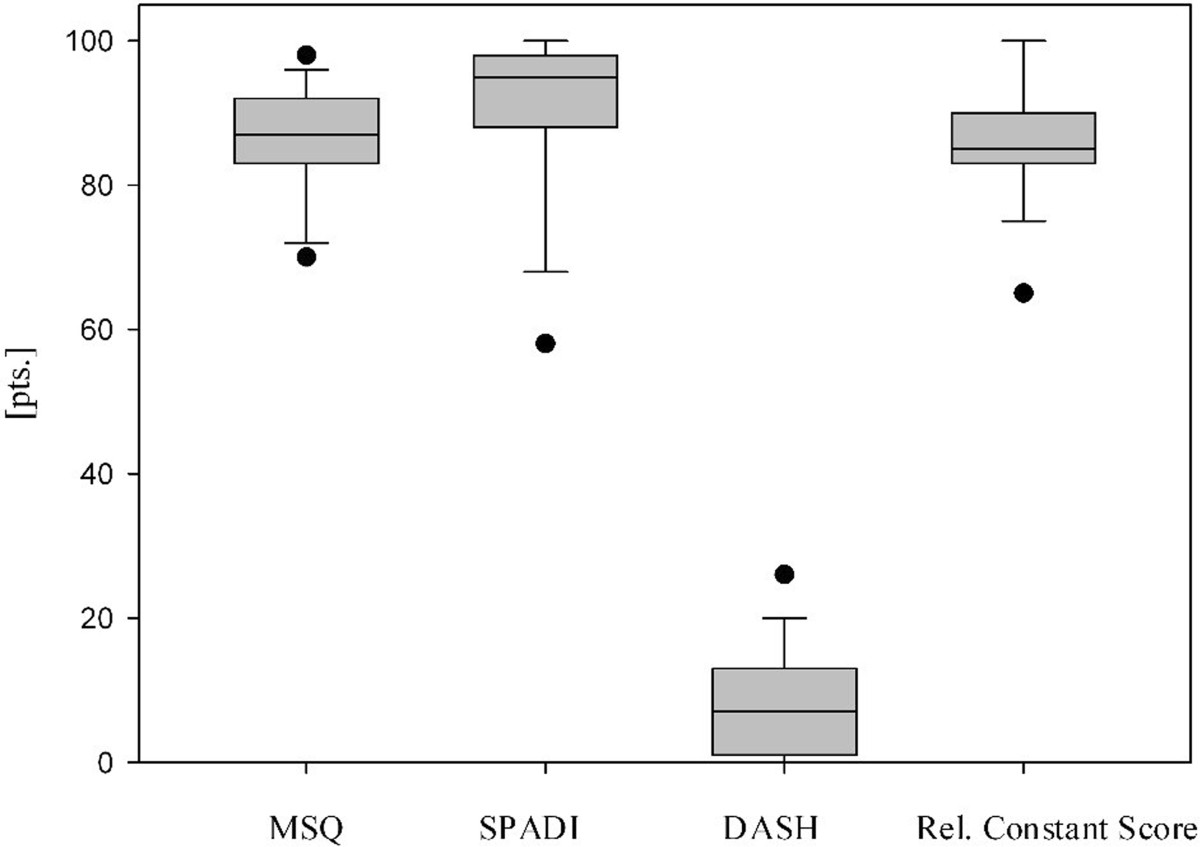
**Patient reported outcomes (Munich Shoulder Questionnaire (MSQ), Shoulder Pain and Disability Index (SPADI), Disability of the Arm, Shoulder and Hand (DASH) score, relative constant score) at a mean follow-up of 14 months.** Data are given as vertical boxplots (median: horizontal boxline; 25–75% interquartile ranges; standard deviations: horizontal line).

**Figure 2:**
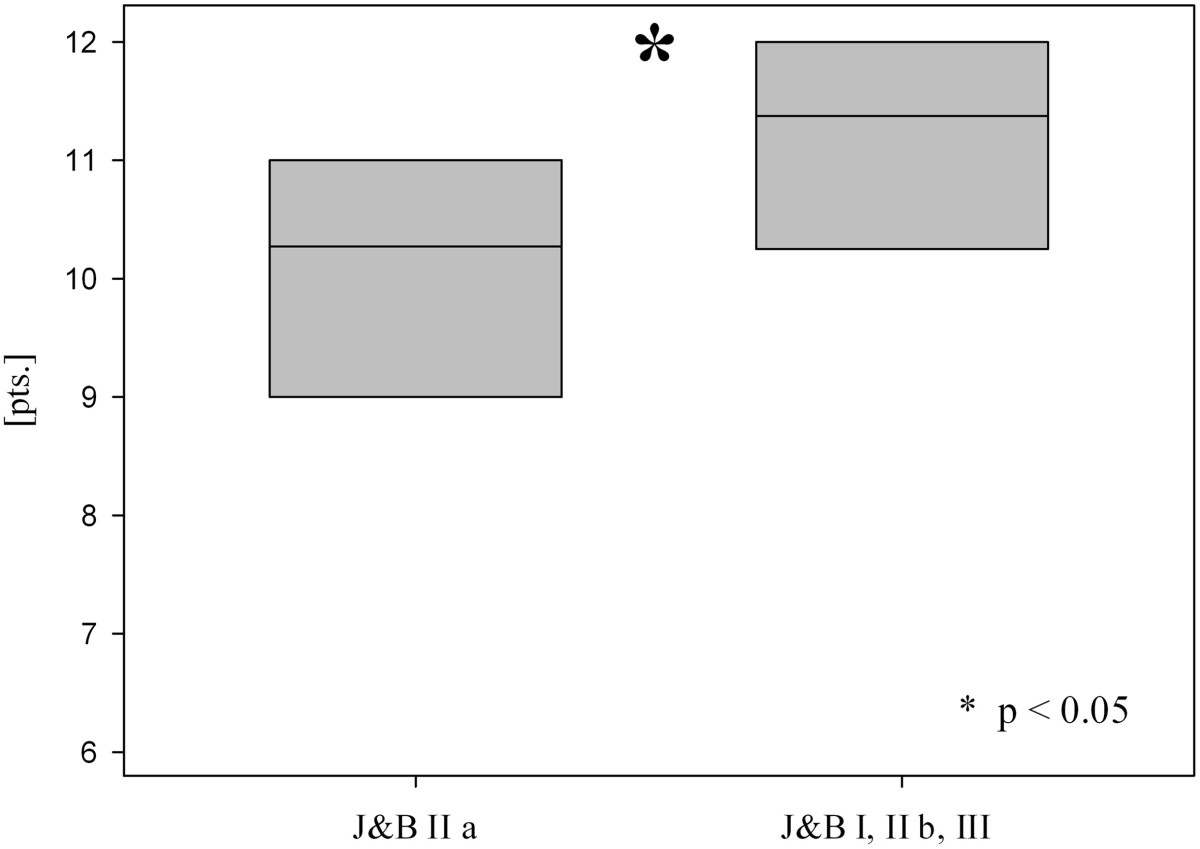
**Mean Taft Score of lateral clavicle fractures Jäger&Breitner (J&B) II a (Neer II B) versus J&B I, II b and III at a mean follow-up of 14 months.** Data are given as vertical boxplots (median: horizontal boxline; 25–75% interquartile ranges). *p < 0.05 group J&B II a (Neer II B) vs. group J&B I, II b, III; Wilcoxon rank-sum test.

**Figure 3:**
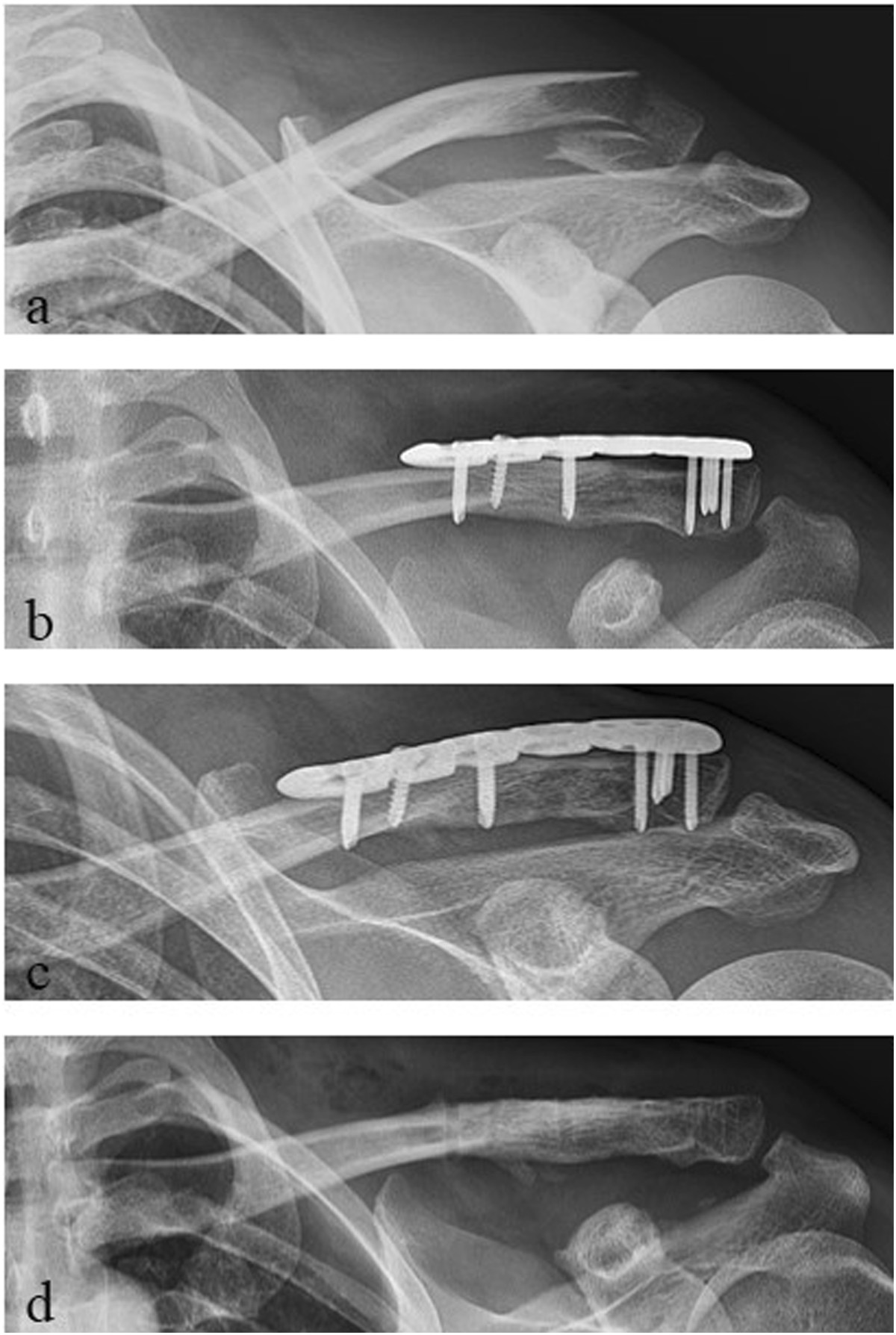
**Radiological outcome of a lateral clavicle fracture J&B II a (Neer II B). a** preoperative; **b** postoperative; **c** 1-year follow-up; **d** after plate removal.

**Figure 4:**
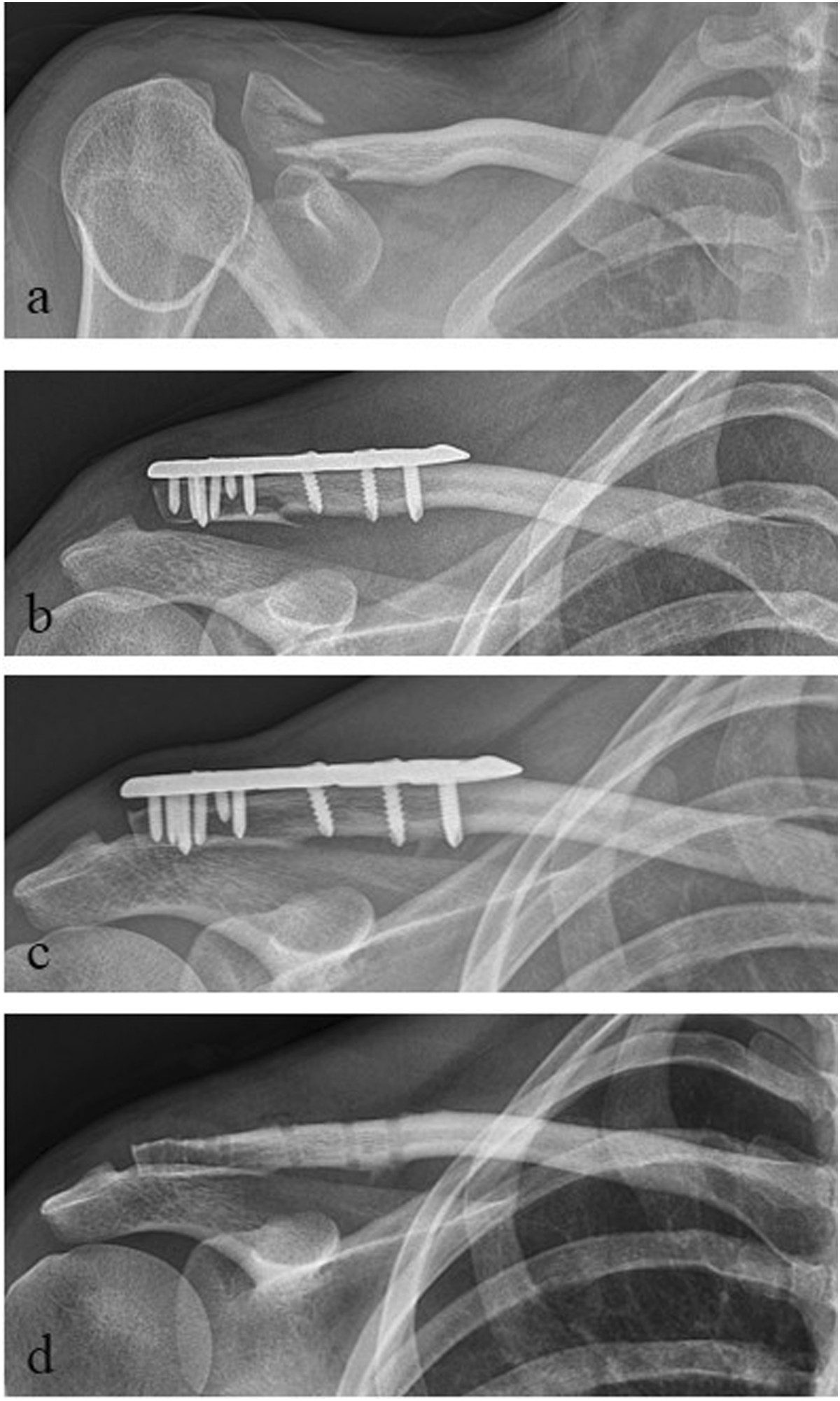
**Radiological outcome of a lateral clavicle fracture J&B II b. a** preoperative; **b** postoperative; **c** 1-year follow-up; **d** after plate removal.

The mean MSQ was 87.0 ± 7.4 points (70–98), the mean SPADI 91.1 ± 11.3 points (58–100), the mean DASH score 7.6 ± 7.3 points (0–26) and the mean normative age- and sex-specific Constant Score 85.6 ± 8.0 points (65–100) (Table 1, Figure 1). The mean Taft Score was 10.7 ± 1.0 points (9–12 points). The mean Taft Score in lateral clavicular fractures with fracture gap between the coracoclavicular ligaments in combination with a rupture of the conoid ligament (J&B II a, Neer II B; n =11) was with 10.3 ± 0.9 points significantly lower than the mean Taft Score of all other types of lateral clavicle fractures (J&B I, II b, III; n =9) which resulted in 11.3 ± 0.9 points (p < 0.05 according to the Wilcoxon rank-sum test; Figure 2). Radiologic bony union occurred in all 20 patients after a mean interval of 6–10 weeks postoperative. Figures 3 and 4 show the radiological outcome of two lateral clavicle fractures (J&B II a and II b).

## Discussion

Treatment of dislocated lateral clavicle fractures, especially of type IIb lesions is controversially discussed [15, 16]. This is due to high non-union rates in conservative and high complication rates in surgical treatment using conventional implants such as the hook plate or k-wire fixation [3]. However, recently developed locking compression plates (LCP) showed promising functional results in small case series [5, 6, 17]. In this prospective clinical trial the results of treating dislocated fractures of the lateral clavicle in 20 patients are reported using a new angular stable implant, the Synthes® LCP superior anterior clavicle plate.

### Demographics

The presented study collective consists of 20 consecutive patients with a mean age of 40.7 ± 11.3 years and a male–female ratio of 14:6 comparable to other outcome studies concerning age and gender [6, 18]. Interpretation of previous case series describing follow-up of locking compression plates for treating lateral clavicle fractures is mostly limited due to small patient collectives (7–14 patients) [5, 6, 19]. Hence, the strength of the obtained results of follow-up examinations in low-incident diseases, such as lateral clavicle fractures, can only provide the fundament and a good starting point for further analysis.

### Complications

No major complications had to be reported in our study. However, 9 of 20 enrolled patients had elective removal of the plate in terms of implant removal. In the current literature, routine removal of metal implants remains a controversial issue [20, 21] with a lack of evident guidelines [22]. Due to the typical surgical risks and complications, implant removal should only be performed in symptomatic patients after a detailed discussion of available treatment options. In our collective, removal of implants was performed in 9 patients due to subjective complaints such as pain if carrying a heavy backpack or sensitivity to weather changes. Compared to other bones of the upper extremity, removal of metalwork is most commonly performed in the clavicle [22], most likely due to the prominent subcutaneous position of the implant especially in athletes with poor soft tissue coverage.

### Functional and radiological outcomes

Since clinical examination by surgeons rating their own patients is characterized by several bias, follow-up assessment was based on a patient-reported outcome questionnaire. The MSQ is a self-administrated and valid questionnaire to assess different aspects of the shoulder function. It allows for a qualitative self-assessment of the SPADI, the DASH score and the Constant Score [12, 13]. In the presented study good to excellent functional results at a mean follow-up of one year after surgery were found. The presented results are comparable to other authors who used a locking compression plate for treating fractures of the lateral clavicle [5, 6, 17]. Advantages of angular stable plate systems in comparison to previous conventional osteosynthetic procedures (such as maintaining sufficient blood perfusion of the periosteum reducing the risk of bony non-union [23]) is one of the reasons for reduced complication rates. The presented results also seem to confirm the promising clinical and radiological outcome of previous smaller case series using the same implant (Synthes® LCP superior anterior clavicle plate) [5, 6].

Fractures of the lateral clavicle are closely anatomically neighbored to the CC ligaments, which are essentially important for the AC stability. Especially a disruption of the more vulnerable, medial conoid ligament can lead to vertical AC instability resulting in a cranialization of the medial clavicle shaft [24]. In our study collective, patients with a lateral clavicular fracture type J&B II a (Neer II B) with disruption of the conoid ligament presented a significant lower postoperative Taft Score compared to all other fracture types according to J&B with intact conoid ligament (I, II b, III; p < 0.05). However the number of patients in the compared groups is relatively low and therefore the reliability of significance is limited. Several authors showed good clinical and radiological results after reconstruction of the CC ligaments in lateral clavicular fractures type J&B II a (Neer II B) which was additionally performed to the actual treatment of the fracture using a locking plate osteosynthesis [6, 17, 25]. Loriaut et al. [26] and Takase et al. [27] reported a satisfactory outcome after single arthroscopic reconstruction of the CC ligaments in unstable lateral clavicle fractures type J&B II a (Neer II B) without plate osteosynthesis. However, biomechanical evaluation showed increased stiffness, higher resistance to compression and decreased displacement of locking plate osteosynthesis combined with CC reconstruction compared to either technique alone [28]. Hence initial evidence is provided that single reconstruction of the CC ligaments in unstable lateral clavicle fractures type J&B II a (Neer II B) could be an insufficient approach due to the remaining instability allowing for fragment movement with an increased risk of pain and non-union.

### Limitations

Despite of the prospective nature of our study it has several limitations to be mentioned. First of all the small number of included patients is considered as limitation. The number of patients in the compared groups is relatively low and therefore the reliability of significance is limited. However the literature does only provide evaluations of the same implant with even lower numbers of patients and therefore we contribute to the poor data situation in literature of this low-incidence-disease with a study of a comperatively high number of patients. A second limitation constitutes the uneven age distribution in both groups regarding AC instability. Although the disruption of the conoid ligament in lateral clavicle fractures J&B IIa presents a relevant reason for postoperative AC instability, we cannot exclude the higher patient age in this group as a substantial confounder. A third drawback of our study is of course that the postoperative rehabilitation was performed on an outpatient basis and thus was not performed in a standardized way. Although physiotherapy should be done according to a standard protocol we cannot guarantee the patient’s compliance.

## Conclusions

By the development of locking compression plates for the treatment of lateral clavicle fractures new perspectives for the so far frustrating surgical therapy have arised. The precontoured Synthes® LCP superior anterior clavicle plate leads to sufficient stabilization and good functional outcome with high patient satisfaction when used for displaced lateral clavicle fractures. In fractures between the coracoclavicular ligaments in combination with a rupture of the conoid ligament (J&B II a, Neer II B) the reconstruction of the CC ligaments additionally to locking plate osteosynthesis showed superior biomechanical stability results. Hence we favor an arthroscopic CC reconstruction additional to open LCP osteosynthesis in lateral clavicle fractures type J&B II a (Neer II B). However, our results still need to be substantiated by analyzing greater patient cohorts which is the focus of our study group.

## Authors’ original submitted files for images

Below are the links to the authors’ original submitted files for images. Authors’ original file for figure 1 Authors’ original file for figure 2 Authors’ original file for figure 3 Authors’ original file for figure 4

## Abbreviations

AC: Acromio-clavicular
CC: Coraco-clavicular
J&B: Jäger and Breitner
ORIF: Open reduction and internal fixation
MSQ: Munich shoulder questionnaire
SPADI: Shoulder pain and disability index
DASH: Disability of the arm, shoulder and hand
LCP: Locking compression plate.
