# Supplementary figures and images for: Clinical and radiological outcome following treatment of displaced lateral clavicle fractures using a locking compression plate with lateral extension: a prospective study
Source: BMC Musculoskelet Disord. 2014 Nov 19;15:380. doi: 10.1186/1471-2474-15-380 (PMC4247764; doi:10.1186/1471-2474-15-380)

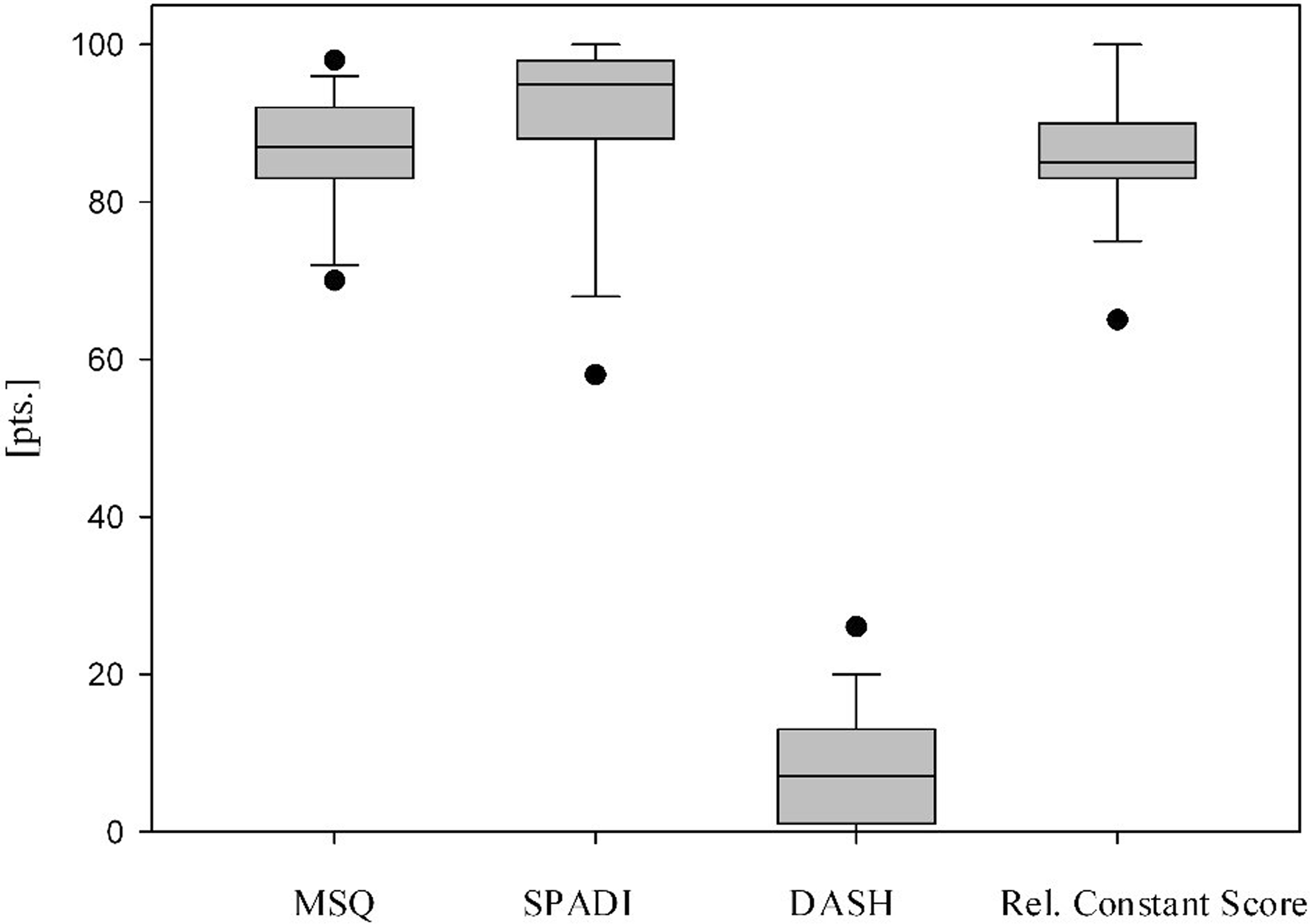

Supplement: Supplementary file 1 — Authors’ original file for figure 1 [file 12891_2014_2331_MOESM1_ESM.tif]

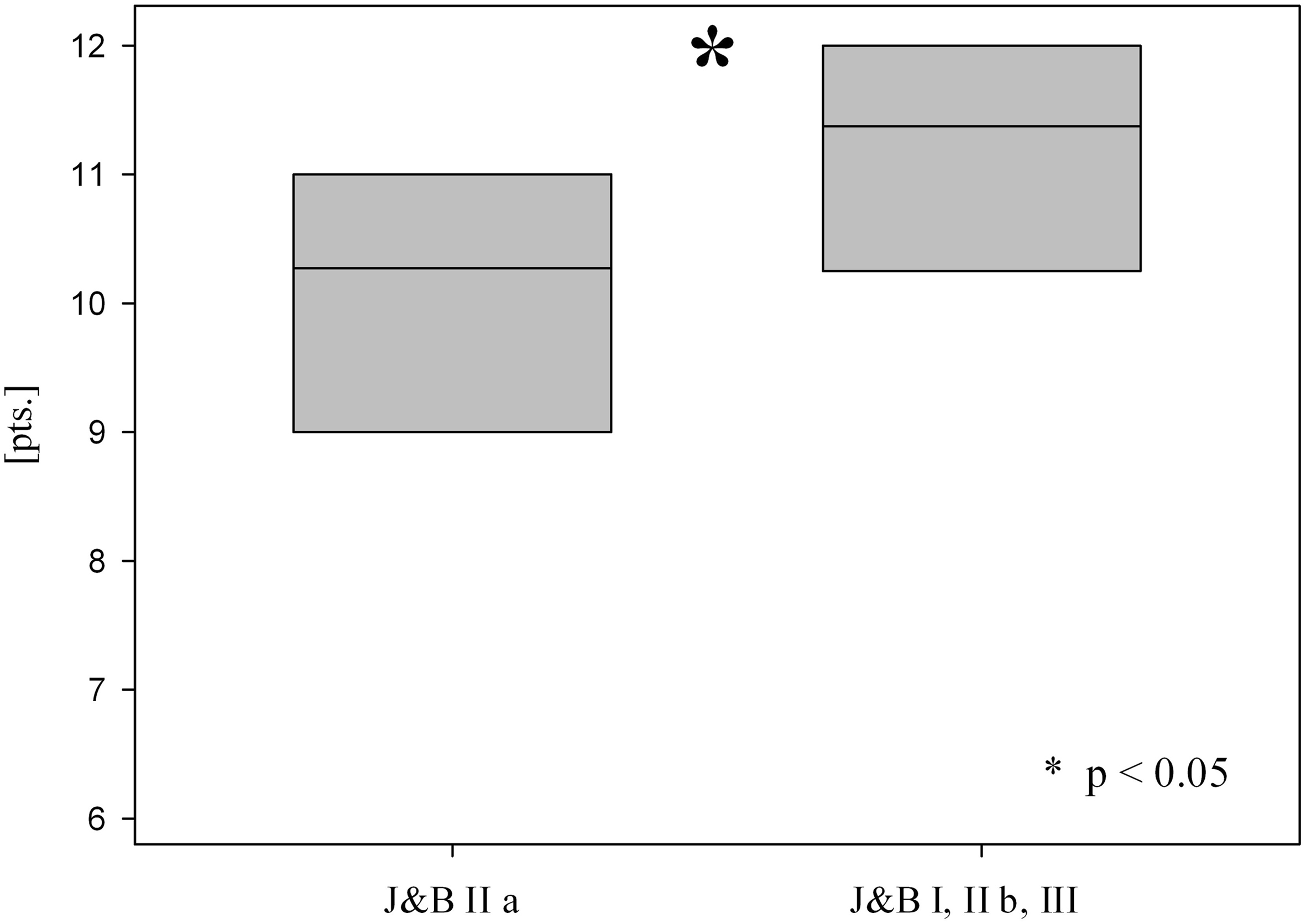

Supplement: Supplementary file 2 — Authors’ original file for figure 2 [file 12891_2014_2331_MOESM2_ESM.tif]

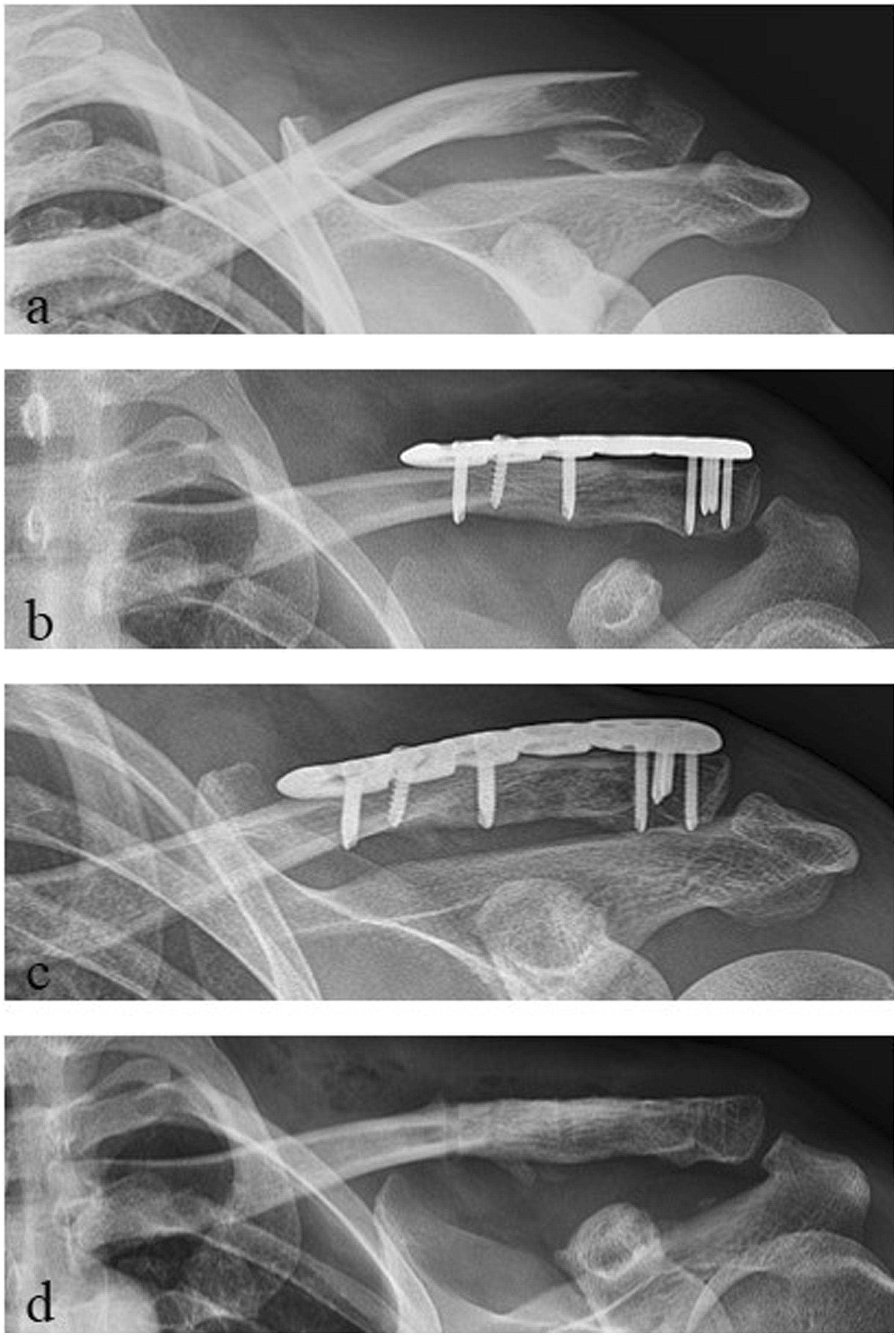

Supplement: Supplementary file 3 — Authors’ original file for figure 3 [file 12891_2014_2331_MOESM3_ESM.tif]

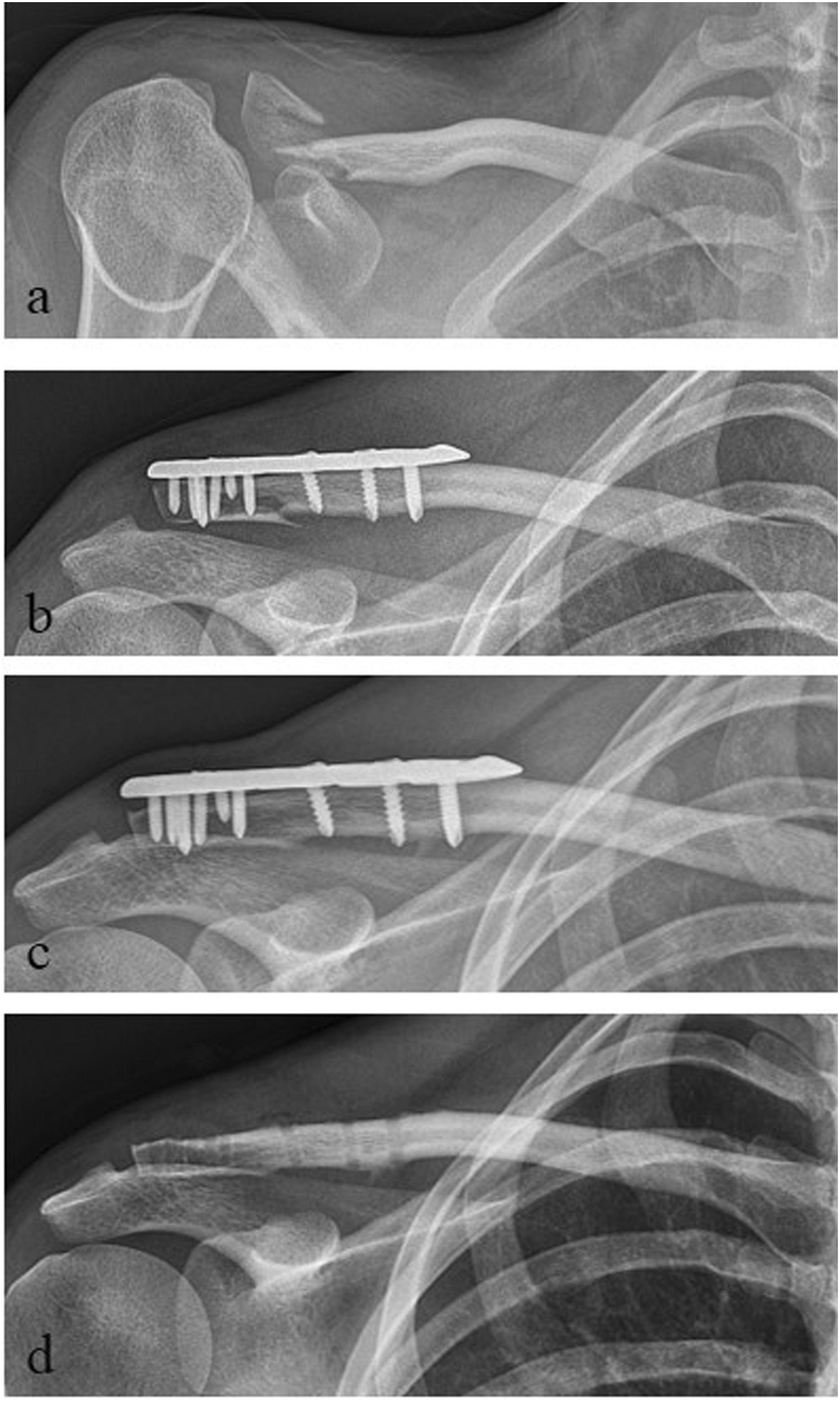

Supplement: Supplementary file 4 — Authors’ original file for figure 4 [file 12891_2014_2331_MOESM4_ESM.tif]
